# A neural substrate for object permanence in monkey inferotemporal cortex

**DOI:** 10.1038/srep30808

**Published:** 2016-08-03

**Authors:** N. C. Puneeth, S. P. Arun

**Affiliations:** 1 Centre for Neuroscience, Indian Institute of Science, Bangalore 560012, India

## Abstract

We take it for granted that objects continue to exist after being occluded. This knowledge – known as object permanence – is present even in childhood, but its neural basis is not fully understood. Here, we show that monkey inferior temporal (IT) neurons carry potential signals of object permanence even in animals that received no explicit behavioral training. We compared two conditions with identical visual stimulation: the same object emerged from behind an occluder as expected following its occlusion, or unexpectedly after occlusion of a different object. Some neurons produced a larger (surprise) signal when the object emerged unexpectedly, whereas other neurons produced a larger (match) signal when the object reappeared as expected. Neurons carrying match signals also reinstated selective delay period activity just before the object emerged. Thus, signals related to object permanence are present in IT neurons and may arise through an interplay of memory and match computations.

We tacitly know that objects in the world persist in space and time. This knowledge – known as object permanence – is present even in infants, suggesting that it is either innate or acquired without explicit instruction^1^,^2^. The classic finding is that infants look longer at displays in which an occluded object changed identity than when it did not^3^. Despite many insights about the nature and progression of this knowledge in infants, we know little about the possible neural substrates.

In the monkey, a likely neural substrate is the inferotemporal (IT) cortex, an area critical for object recognition^4^,^5^,^6^. Importantly, in monkeys trained to perform delayed match-to-sample tasks, IT neurons show selective delay period activity as well as match enhancement and suppression^7^,^8^,^9^,^10^,^11^,^12^. While these are desirable properties for encoding object permanence, they have only been observed after extensive behavioral training on working memory tasks. Thus, we do not yet know whether naïve animals possess the neural substrates required to encode object permanence. In naïve animals, a small fraction of superior temporal sulcus (STS) neurons show selective delay period activity after an object disappears behind an occluder^13^. However, whether single neurons distinguish between the expected versus unexpected appearance of an object has never been investigated previously.

To address these issues, we recorded the responses of IT neurons of two naïve monkeys that viewed displays in which an object was gradually occluded and either the same or a different object was revealed. Our main finding is that a small subpopulation of neurons keeps track of the occluded object and generates match/mismatch signals for the revealed objects.

## Results

### Experiment design

**Figure 1:**
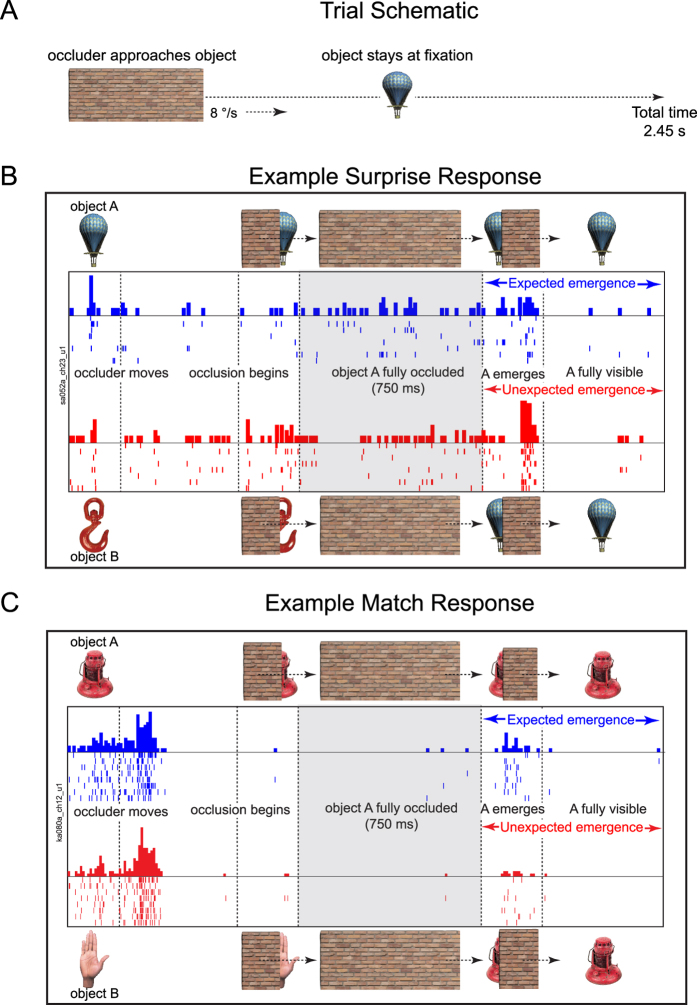
Example surprise and match responses in IT. (**A**) On each trial, an object appeared at fixation and an occluder moved continuously from left to right across the screen and occluded the object when it passed across it. (**B**) Responses of an IT neuron with a surprise signal i.e. higher response for unexpected emergence (*red*) compared to expected emergence (*blue*). Average firing rate (in 20 ms bins) is shown in bars, and tick marks in each row represent the times of individual spikes in a trial. Images above and below the responses show the schematic of the screen content at the corresponding points in the trial. Object images reprinted with permission from Hemera Photo Objects. (**C**) Responses of an IT neuron with a match signal i.e. a higher response for expected compared to unexpected appearance of an object.

Our basic experimental design is illustrated in Fig. 1A. We trained two macaque monkeys to fixate the center of a screen. On each trial, an object appeared at the center of a screen, followed by an occluder that appeared and moved towards the object to occlude it. The occluder moved across the object to reveal either the same or a different object. Animals were simply required to maintain fixation on the center of the screen throughout the trial. The same and different object conditions occurred with equal probability to avoid creating an expectation for either condition. The animals were naïve and never received any other behavioral training. As a result, any expectation generated by the animal can only be due to priors that are either innate or learned through natural experience.

The crucial comparison is between the two visually identical conditions in which (1) an object reappeared as expected after being hidden (expected emergence) and (2) the same object appeared unexpectedly following occlusion of another object (unexpected emergence). Because the two conditions involve identical visual stimuli emerging from behind an occluder, any systematic difference in the neural response to these two conditions can only be attributed to a reflexively generated expectation or violation at the level of single neurons. We tested a number of objects using this design that appeared either as expected or unexpectedly from behind an occluder.

Our goal was to investigate whether signals related to the expectation or violation of object emergence are present at the level of single neurons in IT cortex. But we also wondered whether the animals exhibited any subtle differences in behavior between the two critical conditions. In classic studies of object permanence, human infants look longer at displays with unexpected emergence. Accordingly, in the animals’ behavior, we looked for systematic differences in gaze position or accuracy between the two conditions. However there were none (see Methods). This null result could be a consequence of our design: in the classic studies an object moves behind an occluder, whereas in our experiment, the object stays at fixation. Thus, an unexpected emergence may draw the animals’ attention but need not have a consequence for gaze position or task accuracy.

### Match and surprise signals during occlusion

We recorded the responses of a total of 788 units from the left IT cortex of two macaque monkeys. At each recording site, we selected a preferred and non-preferred object (from a library of 24 objects) for the most visually responsive channel among the 24 simultaneously recorded channels. These two objects (say A & B) were shown in all possible combinations: either A or B was occluded and either A or B was revealed. For the purposes of our analyses however, we compared the expected and unexpected emergence of both objects independently for each neuron (i.e. AA vs BA and BB vs AB). We therefore analyzed a total of 1576 pairs of responses derived from 788 neurons.

We observed two types of neural responses that distinguished between expected and unexpected emergence of objects, as illustrated in Fig. 1. Some cells, such as the neuron in Fig. 1B, showed a larger “surprise” response when an object appeared unexpectedly. Other cells, such as the neuron in Fig. 1C, showed a larger “match” response when an object reemerged as expected. Both surprise and match responses emerged even before the object became fully visible.

To compare neural responses to the expected and unexpected emergence of an object, we performed a statistical test between firing rates across trials in the expected and unexpected emergence conditions during the period following object emergence (i.e. 50–750 ms after emergence; with a criterion α = 0.05, ranksum test). We observed significant response differences in a subset of the pairs (67 of 1576 i.e. 4%). These effects occurred in a small population of neurons (n = 64 cells i.e. 8%). Most of these effects were enhanced “surprise” responses to unexpected emergence (n = 45) whereas the remaining ones (n = 22) were enhanced “match” responses to expected emergence.

**Figure 2:**
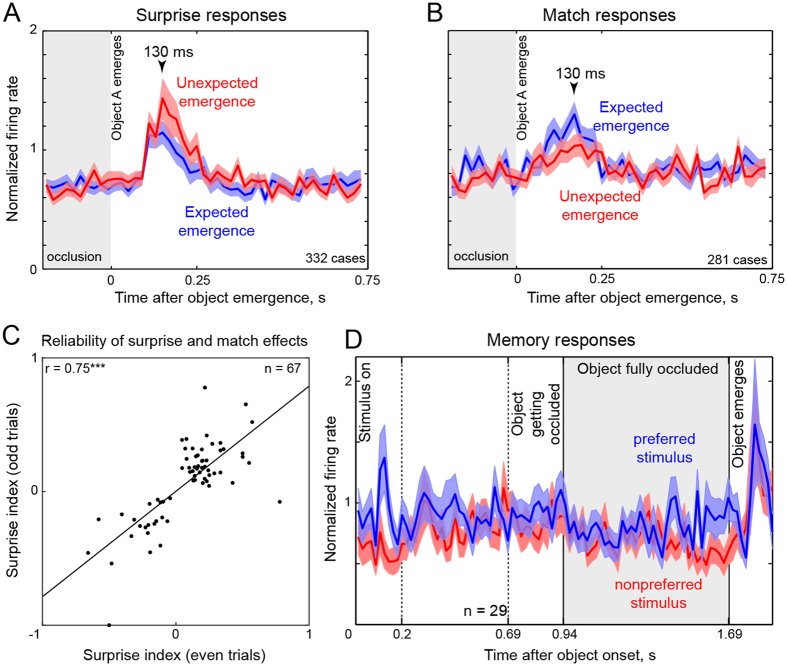
Time course of surprise, match and memory signals in IT neurons. (**A**) Unbiased estimates of the time course of surprise responses. *Blue curves* represent the average normalized response (in 20 ms bins) to expected emergence of an object (blue) across neurons with a significant surprise effect. *Red curves* are the corresponding time courses for the unexpected emergence of the same object. Shaded regions around the solid lines represent standard errors of the mean. The gray region represents the period in which the object was completely hidden behind the occluder. (**B**) Unbiased estimates of the time course of match responses to expected emergence (blue) and unexpected emergence (red). Other conventions are the same as in (**A**). (**C**) Surprise index calculated from odd-numbered trials plotted against surprise index for even-numbered trials across all significant surprise/match effects (n = 67). The positive and significant correlation indicates that the surprise and match effects, whenever they occurred, were highly reliable. (**D**) Average normalized firing rate (in 20 ms bins) for neurons showing a significant memory effect during the last 250 ms of the occlusion period to their preferred stimulus (*blue*) and non-preferred stimulus (*red*). Note that responses to the preferred stimulus (*blue*) shown here are averaged across subsequent emergence of both preferred (expected) and non-preferred (unexpected) stimuli. The same holds for the non-preferred responses (*red*). As a result, the preferred and non-preferred responses overlap completely in the object emergence period.

Although the total number of significant effects detected (4%) is similar to the fraction expected by chance (5%), their division into surprise and match responses (45 surprise, 22 match) was significantly different from the even split expected by chance (p = 0.007, chi-squared test). The preponderance of surprise over match effects was present in both monkeys (numbers of surprise and match effects: 31 & 13 in *Ka*, 14 & 9 in *Sa*) and this difference attained significance in one monkey (p = 0.01 for *Ka*, p = 0.4 for *Sa*). Furthermore, cells that exhibited a significant difference between the two conditions for one object also tended to do so for the other object tested (*r* = 0.13, p < 0.0005 between the p-values for each object comparing the expected and unexpected emergence condition using a rank-sum test). Likewise, the effect was extremely repeatable whenever it occurred: to quantify the repeatability of the surprise/match effects, we calculated a modulation index of the form (U − E)/(U + E) where U and E are the firing rates during the unexpected and expected emergence conditions, after separating the trials of each neuron into two halves. Across the significant surprise effects, this yielded a highly significant positive correlation (r = 0.75, p < 0.00005; Fig. 2C). All these patterns could not have been obtained had our analysis detected only chance fluctuations.

To investigate whether surprise/match effects were more frequent for the preferred object, we counted the number of effects observed for the preferred and non-preferred objects. The number of significant effects were slightly larger for the preferred object (57% i.e. 37 of 65) but this difference was not statistically different from the fraction expected by chance (p = 0.32, chi-squared test). We also found no particular tendency for some objects or object pairs to produce more effects compared to others.

To investigate the time course of the surprise and match effects, we devised a method to obtain unbiased estimates of signal strength. A naïve estimate of surprise signal strength would be to identify pairs in which firing rate was significantly larger in the unexpected emergence condition, and then calculate the firing rate for these pairs in each time bin. However, this method is circular because the same data is being used to identify significant effects and to plot the time course. As a result, the naïve estimate would yield non-zero values for signal strength even for noise. To solve this problem of double dipping, we classified each pair of responses as surprise or match based on one half of the data, and calculated the time course on the other half of data (see Methods). The resulting estimates of the time course of the surprise and match effects are shown in Fig. 2. Both surprise and match signals attained their peak simultaneously (t = 130 ms after object emergence) but showed subtle differences: surprise signals were stronger overall (Fig. 2A) but match signals appeared to rise earlier (Fig. 2B).

Both surprise and match signals can occur only if there is some memory for the occluded object when it is no longer visible. We therefore set out to investigate whether neurons show selective delay period activity for the occluded object. To this end, we compared the responses of each cell during the last 250 ms of the occluded period between conditions in which either object A or B was occluded. A small population of neurons (n = 29) showed a significant difference in response (p < 0.05, ranksum test). Again, given the small number of neurons with a memory signal, we were concerned that we were detecting chance fluctuations. However, cells with chance fluctuations would show no consistent relationship between their preferred object in the visual period with the firing rate during the memory period. Alternatively, if these cells carried memory signals for their preferred stimulus, they will show consistently greater activity following occlusion of their preferred stimulus compared to the non-preferred stimulus.

To assess these possibilities, we calculated the average normalized responses of these cells to their preferred and non-preferred stimuli (as determined during the visual response period) for the entire trial up to the point of object emergence. In this analysis, any response differences during the delay period that are not systematically related to the visually presented objects will average out to zero. The resulting plot (Fig. 2D) shows that selective delay period activity is absent immediately after occlusion but is reinstated towards the end of the occlusion period just as the object is about to emerge.

**Figure 3:**
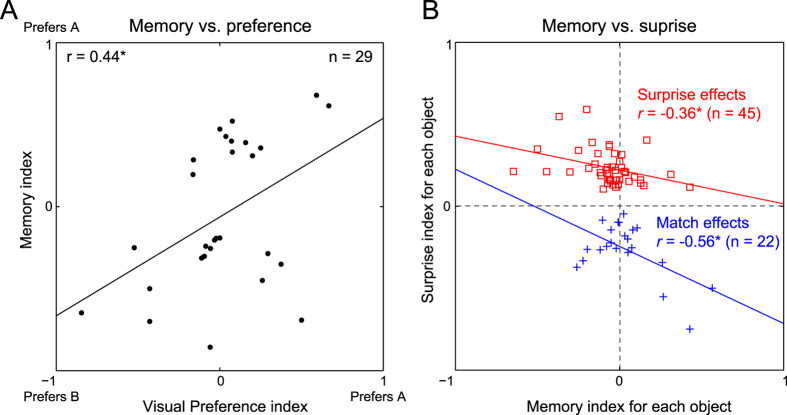
Memory signals during occlusion conditions. (**A**) Memory index plotted against visual preference index for neurons showing a significant memory effect (n = 29). (**B**) Surprise index for each object plotted against the memory index for that object in the occlusion conditions, for significant surprise effects (n = 45, *red squares*) and significant match effects (n = 22, *blue crosses*).

To further confirm that selectivity in the occlusion period is related to selectivity for the visible object, we calculated modulation indices of the form (A − B)/(A + B) during the object presentation period (50–200 ms after onset) and during the last 250 ms of the occlusion period, where A and B are the responses to the two objects. For cells that showed significant memory effects, memory modulation showed a significant correlation with object selectivity (r = 0.44, p = 0.019; Fig. 3A). We conclude that a small fraction of IT neurons shows selective delay period activity that is reinstated close to the time of object emergence.

If delay period activity is being reinstated close to the object emergence period, we would expect this memory signal to become stronger as the animals learned to anticipate the emergence of the object over the course of the recording sessions. To investigate this possibility, we quantified the selective delay period activity using a memory index of the form |A − B|/|A + B| and asked whether the strength of the memory signal became stronger in the second half of the recordings compared to the first half. The delay period activity was more selective in the second half compared to the first half, but this difference was not statistically significant (average memory index: 0.39 during the first half, 0.44 during the second half, p = 0.55, unpaired t-test). Thus, at least with this level of analysis, we do not see an increasing trend of selective delay period activity over the course of our recordings.

Do cells that show surprise/match signals also carry memory signals ? To address this question, we first quantified the strength of the surprise/match effect for each object using a surprise index of the form (U − E)/(U + E), where U and E are the firing rates in the unexpected and expected conditions respectively (during the 50–750 ms period following object emergence). A negative surprise index would occur if E > U, and therefore represents a match response. For each object we calculated a memory signal strength by calculating the modulation index in its favor i.e. (A − B)/(A + B) where A is the object of interest and B is the other object tested for that neuron. For all significant surprise/match effects, we plotted the surprise index against the memory index (Fig. 3B). This yielded a significant negative correlation for match effects (r = −0.56, p = 0.007). Note that the surprise index will be negative for match effects. Thus, the negative correlation here implies that the stronger the memory signal for a particular object, the stronger is the match effect for that object. For surprise effects also we observed a significant negative correlation (r = −0.36, p = 0.015). To measure the robustness of the reported correlations, we performed a bootstrap analysis: we sampled the data with replacement each time and calculated the correlation coefficient. The resulting bootstrap-resampled correlations were positive only 4% of the time for the surprise effects and only 4.5% of the time for the match effects. These correlations were also present in both monkeys (monkey *Ka*: r = −0.40, p = 0.02 across 31 surprise effects; r = −0.47, p = 0.11 across 13 match effects; monkey *Sa*: r = −0.30, p = 0.3 across 14 surprise effects; r = −0.75, p = 0.02 across 9 match effects).

Thus, the stronger the memory signal for a particular object, the weaker the surprise effect. Both negative correlations lead to the same conclusion: memory signals tend to occur in cells with match signals but not in cells carrying surprise signals.

The fact that the monkeys were exposed to unexpected and expected trials over the course of the experiment raises the possibility that the animals may have learned to “expect the unexpected”. To test this possibility we took the absolute value of the surprise index for all significant effects (n = 67) and separated them into those observed during the first half of our recordings and those observed in the second half. If the animals were becoming habituated to the unexpected emergence, this would predict a smaller absolute surprise index in the second half. However, there was no significant drop in the magnitude across the two halves of our recordings (median absolute surprise index: 0.21 for the first 33 effects, 0.23 for the second half, p = 0.7, Wilcoxon’s rank-sum test).

### Is occlusion necessary for the generation of match/surprise signals?

The above surprise and match signals could in principle have been observed even in the absence of an occluder, by sheer virtue of the fact that the same stimulus is repeated after a short delay. This is expected because IT neurons show reduced responses to repeated presentations of objects, a phenomenon known as repetition suppression^14^,^15^,^16^,^17^,^18^,^19^,^20^. To investigate this possibility, we included an additional set of fixation control conditions in the main experiment. Although we initially considered using an invisible occluder, we rejected this possibility because it too could produce the percept of occlusion. We therefore chose a control condition in which there was no moving stimulus. The fixation control trials were identical in all respects to the occlusion trials except that there was no occlusion. Instead, the object was replaced by a fixation dot at the exact time at which the occluder contacted the object in the occlusion trials (i.e. 700 ms after the object turned on) and stayed on for 1000 ms. Following this it was replaced by either the same or different object as before. Thus the time for which objects were fully visible was identical in the occlusion and fixation control trials. However, since at least some portion of the object was visible during the occlusion trials, we predicted that repetition suppression would (if at all) be stronger in the occlusion trials.

**Figure 4:**
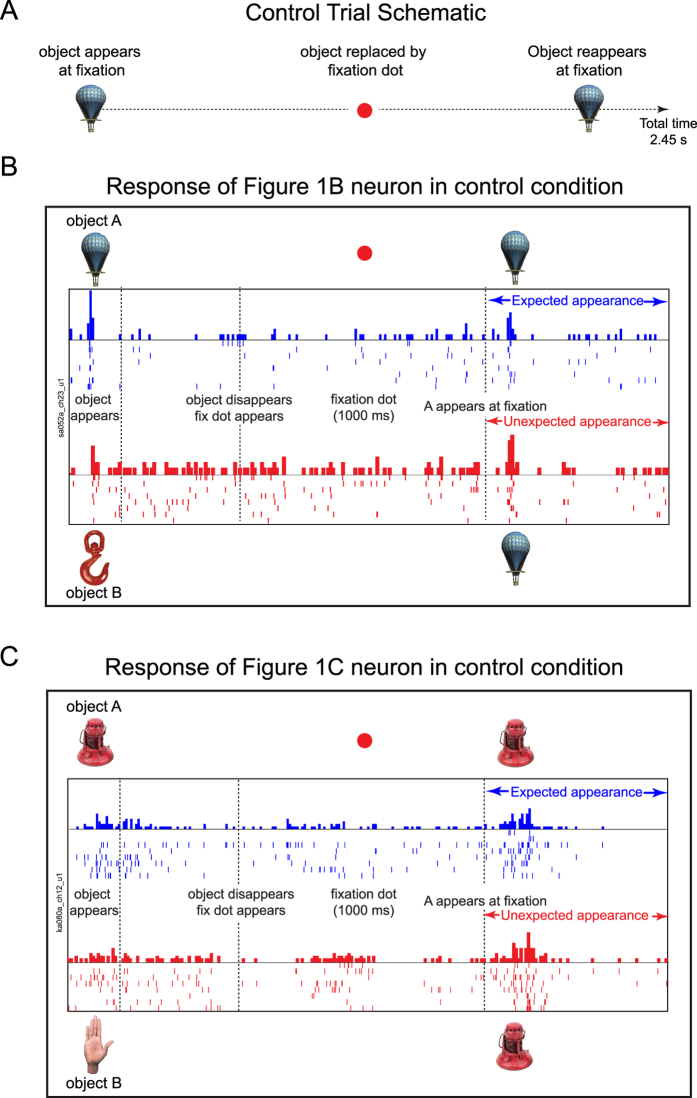
Example responses in the fixation conditions. (**A**) On each trial in the fixation control conditions, an object appeared at fixation and was replaced by a fixation dot, followed by either the same or different object. (**B**) Responses of the Fig. 1B neuron in the fixation control conditions. All conventions are identical to Fig. 1. Object images reprinted with permission from Hemera Photo Objects. (**C**) Responses of the Fig. 1C neuron in the fixation control conditions.

We repeated all the data analyses on this condition and the salient results are presented here. Figure 4 illustrates the responses of the neurons in Fig. 1 during the fixation control trials. The neuron of Fig. 1A – which showed a strong surprise signal in the occlusion condition – showed no difference between the equivalent fixation conditions (Fig. 4A). Likewise, the neuron of Fig. 1B – which showed a match signal – showed no difference during the equivalent fixation conditions (Fig. 4B).

**Figure 5:**
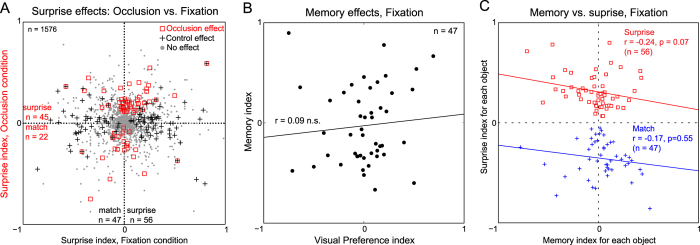
Surprise and match signals in occlusion and fixation conditions. (**A**) Surprise index for the occlusion condition plotted against surprise index for the fixation control conditions (2 pairs/neuron × 788 neurons = 1576 data points). *Red squares* and *black crosses* indicate response pairs with a significant effect (p < 0.05) in the occlusion and fixation conditions respectively. *Gray dots* indicate response pairs with no significant effect. (**B**) Memory index plotted against the visual preference index for neurons with significant memory effects. (**C**) Surprise index for each object plotted against the memory index for that object in the fixation conditions, for significant surprise effects (n = 56, *red squares*) and significant match effects (n = 47, *blue crosses*).

To compare the match and surprise signals in the occlusion and fixation conditions, we calculated a surprise index for each object that could range from −1 (for pure match) to +1 (for pure surprise) in the occlusion and fixation conditions and plotted one against the other (Fig. 5A). We found no correlation between the surprise indices in the two conditions (r = 0.01, p = 0.76 across all 788 × 2 = 1576 objects). While there were 67 pairs (4.3%) that showed a significant difference in the occlusion conditions, there were 108 pairs (6.8%) with a significant difference in the fixation conditions. This difference could reflect intrinsic differences between the nature of the stimuli in these two conditions which were not equated in any way. We therefore focused instead on comparing the pattern of results in the two conditions.

We started by asking whether significant effects in the occlusion conditions tended to co-occur with significant effects in the fixation condition. There were only 8 instances in which a significant difference between expected and unexpected emergence occurred in both the occlusion and fixation conditions. This pattern was not significantly different from the number expected if the two effects were distributed independently (expected overlap: 1576*0.068*0.043 = 4.6 pairs; p = 0.2, chi-squared test). The distribution of significant effects was also different in the two conditions: in the occlusion trials there were 45 surprise and 22 match effects, and this split was significantly different from the 50–50 split expected by chance (p = 0.007, chi-squared test). In contrast, in the fixation trials, there were 56 surprise and 47 match effects, and this split (54% surprise) was not significantly different from the chance outcome (p = 0.43, chi-squared test). Finally, the distribution of surprise and match effects in the occlusion trials was significantly different from the distribution in the fixation trials (45/22 during occlusion versus 56/47 during fixation: p = 0.0001, chi-squared test).

Next we asked whether cells show any memory signals during the fixation conditions. To this end, we first compared firing rates of each neuron during the last 250 ms of the occlusion period. A total of 47 cells showed a significant effect (p < 0.05, ranksum test). To investigate the relationship between visual selectivity and delay period activity, we plotted the memory preference index for cells with a significant memory effect against their visual preference index as before. This plot yielded no significant correlation (r = 0.09, p = 0.55; Fig. 5B). Finally, we asked whether there is a consistent relationship between memory signals and surprise/match signals in the fixation trials. As before, we plotted the surprise index for each object in the fixation condition against the memory index for the same object during the last 250 ms of the fixation period. We observed no significant correlation between surprise and memory signals (r = −0.24, p = 0.07; Fig. 5C) and between match and memory signals (r = −0.17, p = 0.55; Fig. 5C). Thus, there is no systematic relationship between memory and surprise/match signals in the fixation conditions.

To summarize our findings in the fixation control conditions: (1) there was no systematic relationship between surprise/match effects in occlusion and fixation conditions; (2) for the significant effects observed in the fixation condition, we saw no systematic memory signals and no systematic relationship between memory and match signals as we did in the occlusion conditions. Thus, IT neurons show qualitatively different responses to the occlusion and fixation conditions. We conclude that the surprise/match signals in the occlusion trials cannot be attributed to the simple repetition of stimuli in time, but are likely to be specific to the percept of occlusion.

## Discussion

Here we have shown that a small population of IT neurons carry signals that track the identity of an occluded object and generate match/mismatch responses to the object revealed after occlusion. These signals are present in two groups of cells: (1) One group of cells reinstate selective activity for the occluded object just before the object is revealed, followed by a “match” signal when the same object reappeared as expected; (2) A relatively larger group of cells that produces a surprise signal when the object appeared unexpectedly. These observations suggest that IT neurons carry signals related to encoding object permanence. Below we review our findings in the context of the existing literature.

The fact that a small subset of IT neurons carry the signals required for object permanence does not imply that they play a causal role. Another area may show more prevalent effects and the relatively fewer effects in IT may result from sparse feedback from that area. For instance, both prefrontal and perirhinal cortex – which are connected to IT – show stronger match/mismatch effects than IT^21^,^22^. On the other hand, their prevalence in IT may be higher when tracking is behaviorally relevant, such as when food is occluded. These signals may still not occur in a majority of IT neurons though: for instance, the estimated fraction of neurons with match/nonmatch effects in animals performing match-to-sample tasks range from 30% in IT^11^ to 48% in perirhinal cortex^8^,^9^,^10^. More generally, several other areas may participate in tracking objects that are no longer visible. For instance, STS neurons show selective delay period activity after objects disappear behind an occluder^13^. Dorsal stream areas such as MT and FEF keep track of object motion behind occluders^23^,^24^,^25^ and premotor areas respond to objects in peripersonal space even in complete darkness^26^. However, whether these areas encode surprise or match signals has not been explicitly tested.

Our finding that only 4% of the sampled IT neurons contain surprise/match signals during object occlusion raises the possibility that we were simply detecting chance fluctuations. We consider this possibility unlikely for several reasons: (1) Chance fluctuations should have produced equal numbers of surprise and match effects, but the proportion observed was significantly different from that expected by chance; (2) Chance fluctuations should have produced inconsistent effects across trials, whereas in fact the effect strength was extremely consistent across trials whenever the effect occurred; (3) Chance fluctuations should have produced no systematic relationship between the memory signal and selectivity during the visual period, but in fact there was a clear correlation (Figs 2C and 3A); (4) Chance fluctuations should have produced no correlation between surprise/match signal strength with memory signal strength, but this was not the case either (Fig. 3B). Instead, these results indicate the presence of a small subpopulation of IT neurons that may be part of a functionally specialized circuitry for the processing of match/nonmatch signals.

In addition to the above arguments, there is a philosophical problem with detecting small specialized subpopulations of neurons within a larger neural population. Consider an experiment in which an electrode array was improperly implanted such that only a few electrodes were in visual cortex, and a statistical test was performed to detect visually driven activity for each neuron. The finding that only 5% of the neurons were visual according to such a test would hardly be grounds to reject them as “chance fluctuations”. It would be equally absurd – in correcting for multiple comparisons – to require that these cells need to be increasingly more significantly visual depending on how many *other* non-visual units were present. The problem with this kind of argument is that the statistical tests are assumed to sample a homogeneous population, whereas the underlying population could well be heterogeneous – in which case the statistical argument is no longer valid. Thus, our detection of a small subpopulation of IT neurons with surprise/match signals is not necessarily diminished by the finding of a larger population of neurons that do not carry these signals, particularly when the detected effects are highly consistent and inter-related.

Our finding that IT neurons show memory and match/nonmatch signals is consistent with similar findings in animals performing delayed match-to-sample tasks^7^,^8^,^9^,^10^,^11^,^12^,^27^. Our results provide several new insights into the nature of match and nonmatch signals: First, we found that memory and match signals are related, suggesting that match signals may lead to surprise signals. Second, memory and match/nonmatch signals in the occlusion and fixation conditions were qualitatively different. Third, surprise effects were far more prevalent in the occlusion condition than in the control trials, even though the control trials had a smaller inter-stimulus interval (which should have produced larger repetition suppression effects). We speculate that the preponderance of surprise effects could be because of attention directed towards unexpected emergence compared to expected emergence, but establishing this will require further study. These findings raise the general question: under what conditions do IT neurons keep track of objects that are no longer visible? Although we initially considered using an invisible occluder as a possible control, we did not include this condition because it produced an equally strong percept of occlusion. We speculate that any condition that produces a strong percept of occlusion might automatically engage match/nonmatch signals in the brain.

Finally, the signals we have observed can be interpreted as a form of the transitional surprise signal reported in IT neurons^28^. The fact that specific image associations are learned by IT neurons even during passive viewing has been shown elegantly by a number of studies^28^,^29^,^30^,^31^. However, the fact that we have observed object permanence signals for an arbitrarily selected set of objects suggests that this signal cannot be specific to any particular object. Precisely how spatiotemporal regularities or paired associations generalize from one specific instance to a wider set of possibilities remains an important open question. In human infants, it is not clear whether object permanence is innate, but these priors certainly mature systematically with age^1^,^2^. We propose that neuronal circuitry in IT cortex consists of generalized circuitry to encode a variety of spatiotemporal regularities in natural vision.

## Methods

All experiments were performed according to an experimental protocol approved by the Institutional Animal Ethics Committee of the Indian Institute of Science, Bangalore and the Committee for the Purpose of Control and Supervision of Experiments of Animals, Government of India. Most experimental methods are described in detail elsewhere^32^ and are summarized briefly here.

### Neurophysiology

Two adult male monkeys (*Macaca radiata*, laboratory designations *Ka* and *Sa*, both aged ~7 years) were used in the study. Prior to recording, each monkey was implanted with a titanium headpost and a recording chamber (Crist Instruments, USA). The recording sites corresponded to the anterior-ventral inferotemporal cortex. The average location of recorded sites was verified using structural MRI to be at anterior +14 mm, lateral +13 mm in monkey *Ka* and anterior +19 mm, lateral +15 mm in monkey *Sa*. Eye movements were monitored using an infrared eye tracker (ETL250, ISCAN Inc). Stimuli were displayed on a 120 Hz LCD monitor (VX2268wm, Viewsonic) that was under the control of a computer running NIMH Cortex. On each day of recording, a 24-channel microelectrode (Uprobe, Plexon Inc, 100 μm inter-electrode spacing along the shank) was inserted through a stainless steel guide tube, and advanced until phasic visual responses were observed on at least one channel. All waveforms were stored and subsequently sorted into single and multiunits using commercial spike-sorting software (Offlinesorter, Plexon Inc). Waveforms that formed visually distinct clusters were sorted as single units whereas those with multiple spikes were sorted as multiunits. For each recording site, we selected the best isolated visual unit across all channels, and selected a preferred and non-preferred object from a library of 24 objects chosen from Hemera Photo Objects. In all we recorded from a total of 45 sites (24 channels/site, 25 sites from monkey Ka, 20 sites from monkey Sa). We occasionally encountered nearby channels that picked up the same action potential. To remove these repeated units, we calculated a shuffle-corrected cross-correlogram for channel pairs recorded in a given site, and removed the smaller-amplitude unit in every pair that showed a cross-correlation larger than a cutoff value of 0.4 at zero lag. Out of a total of 804 units, we removed 16 correlated units using this procedure. This yielded a total of 788 units (644 single units, 144 multiunits; 487 units from *Ka*, 301 from *Sa*). Subsequent analyses revealed qualitatively similar effects for both single unit activity and multiunit activity, so the data presented in the figures include both types of activity. There was also no tendency for surprise, match or memory effects to be present at specific anatomical locations within our recorded sites.

### Occlusion trial design

On each occlusion trial, a small fixation dot (0.05° radius) appeared, followed by an object (measuring 2° wide by 4° high) that was presented for 200 ms. Then an occluder (a brick wall, 2° wide by 8° tall) appeared at a location 8° in the left visual periphery and began to move horizontally towards the right at a speed of 8°/s (Fig. 1A). The location and speed of the occluder were set to contact the object 500 ms after it began to move (the actual timing markers revealed this number to be 490 ms), and completely occluded it 250 ms later. The object was completely occluded for 750 ms, after which the occluder began to reveal the object again. The object was fully visible 250 ms after it began to emerge, and then stayed visible for 500 ms while the occluder moved to a final location of 8° in the right visual periphery. In all, there were four types of conditions involving the two objects (say A & B) chosen for the recording session: either A or B could be occluded and either A or B could emerge from behind the occluder. Each condition was repeated 8 times, resulting in a total of 32 occlusion trials. To familiarize the animals to the occlusion trial design, each monkey was exposed to an average of 200 trials of expected emergence involving all 24 objects used subsequently for recordings. Both monkeys quickly learned (over 2–3 days of 100 trials each) to avoid looking at or tracking the occluder, and began to maintain their gaze on the center throughout.

### Fixation trial design

To assess whether occlusion was necessary for observing surprise/match signals, we included an additional fixation control trials. These trials were identical in all respects to the occlusion trials except that there was no occlusion. Instead, the object was replaced by a fixation dot at the exact time at which the occluder contacted the object in the occlusion trials (i.e. 700 ms after object on) and stayed on for 1000 ms. Following this it was replaced by either the same or different object as before. Thus, the time for which objects were fully visible was identical in the occlusion and fixation control trials. There were a total of four conditions as before: either A or B turned on first, and either A or B appeared second. Each condition was repeated 8 times, resulting in a total of 32 fixation control trials.

### Behavior

Each animal performed a fixation task involving 32 occlusion and 32 fixation control trials. Occlusion and fixation trials were randomly interleaved. Each trial lasted 2.45 seconds, and animals received a juice reward for maintaining fixation throughout within a 4° by 4° window (i.e. half the width of the occluder), failing which the trial was aborted immediately and repeated after a random number of other trials. Although we used a liberal fixation criterion, a post-hoc analysis of eye movements revealed that the animals’ gaze stayed close to the center throughout the trial (mean gaze location relative to center: −1.15° and 0.21° along x and y; average standard deviation across the trial along x and y: 0.53° and 0.38° respectively). Both animals performed the task at 93% correct on average. Their overall accuracy was higher for fixation control trials (average accuracy: 98%) than for occlusion trials (88% on average). Importantly, there were no systematic differences between expected and unexpected emergence trials (average accuracies: 88% for expected emergence, 87.5% for unexpected emergence). Eye movement data also revealed no systematic difference in gaze location between these two conditions.

### Normalization of firing rates

We have used normalized firing rates in all our analyses to equate effect strengths across single- and multi-unit activity. To calculate the normalized firing rate, we divided the raw firing rate for each cell by the maximum average firing rate evoked during the entire trial across all occlusion and fixation conditions. The average firing rate during the entire trial is generally small because most cells have low firing rates during the delay period, and as a result the normalized firing rate during the onset of a visual stimulus typically exceeded 1.

### Correlation coefficients

Throughout, we have used the Pearson’s correlation coefficient which takes into account the covariation in magnitude and is valid for linear relationships. Its validity can be confirmed in Fig. 3 where the relationships look largely linear. More generally, we obtained similar results throughout on using the Spearman’s rank order correlation instead of the Pearson’s correlation.

### Unbiased estimates of surprise and match time courses

Estimating the time course of surprise and match signals is nontrivial for the following reason: the simple-minded approach of identifying all surprise or match effects, and calculating their time course yields nonzero (biased) signal estimates even for random noise because the same data is used to identify the effects and to calculate their time course. To avoid this problem, we used a split-half approach: we used the odd-numbered trials to perform a statistical test on the firing rates (in a 50–750 ms window after object emergence) in the expected and unexpected emergence conditions, and then used the even-numbered trials to calculate the firing rates in each time bin (normalized to the maximum firing rate in the entire trial across all conditions) for those two conditions. We repeated this process again, this time using even-numbered trials for the statistical comparison and the odd-numbered trials for the time course. We then averaged these two estimates to obtain unbiased time courses for the surprise and match signals. If one were to use this approach on random noise, a surprise effect in one half of trials will produce roughly equal responses to both conditions in the other half of trials, resulting in a zero overall contribution to signal strength. We further validated this procedure using a shuffle control which yielded no differences in firing rates.

Because these estimates are based on only half the data, the pairs that were formerly statistically significant using the entire data were now significant only at a level of p < 0.3 on average. We had to estimate this cutoff because the relationship between the p-value and sample size is nonlinear. However, the results were fundamentally unaltered on varying this cutoff. Using this criterion, we obtained 332 time courses for surprise effects and 281 for match effects from a total of 3152 time courses (788 cells × 2 stimuli × 2 estimates/stimulus). These are the numbers reported in Fig. 2A,B. Because each cell contributes 4 pairs to this analyses, ~83 cells contributed to the surprise signal estimates and ~70 cells to the match signal estimates in Fig. 2A,B.

### Unbiased estimates of the memory time course

To obtain unbiased estimates of the memory signal, we first compared the firing rates during the last 250 ms of the occlusion period (when the object was fully occluded) for the conditions in which object A was occluded versus when object B was occluded. Having identified these cells (n = 29, p < 0.05 on a ranksum test during the last 250 ms of the occlusion period), we then plotted their normalized response to the preferred stimulus and non-preferred stimulus (as identified by the one with the larger firing rate in a window 50–200 ms after object onset). Because the memory effect and the object preference were calculated in completely independent time windows, any neural responses in which the memory period response is not systematically related to the visual response will average out to zero.

## Additional Information

**How to cite this article**: Puneeth, N. C. and Arun, S. P. A neural substrate for object permanence in monkey inferotemporal cortex. *Sci. Rep*. **6**, 30808; doi: 10.1038/srep30808 (2016).
